# Multi-state modelling of heart failure care path: A population-based investigation from Italy

**DOI:** 10.1371/journal.pone.0179176

**Published:** 2017-06-07

**Authors:** Francesca Gasperoni, Francesca Ieva, Giulia Barbati, Arjuna Scagnetto, Annamaria Iorio, Gianfranco Sinagra, Andrea Di Lenarda

**Affiliations:** 1 MOX-Modelling and Scientific Computing, Department of Mathematics, Politecnico di Milano, Milano, Italy; 2 Department of Medical Sciences, Università di Trieste, Trieste, Italy; 3 Cardiovascular Center, Trieste, Italy; 4 Cardiology Unit, Papa Giovanni XXIII Hospital, Bergamo, Italy; 5 Cardiovascular Department, Azienda Sanitaria-Universitaria Integrata Trieste ‘ASUITS’, Trieste, Italy; Universita degli Studi di Napoli Federico II, ITALY

## Abstract

**Background:**

How different risk profiles of heart failure (HF) patients can influence multiple readmissions and outpatient management is largely unknown. We propose the application of two multi-state models in real world setting to jointly evaluate the impact of different risk factors on multiple hospital admissions, Integrated Home Care (IHC) activations, Intermediate Care Unit (ICU) admissions and death.

**Methods and findings:**

The first model (model 1) concerns only hospitalizations as possible events and aims at detecting the determinants of repeated hospitalizations. The second model (model 2) considers both hospitalizations and ICU/IHC events and aims at evaluating which profiles are associated with transitions in intermediate care with respect to repeated hospitalizations or death. Both are characterized by transition specific covariates, adjusting for risk factors. We identified 4,904 patients (4,129 de novo and 775 worsening heart failure, WHF) hospitalized for HF from 2009 to 2014. 2,714 (55%) patients died. Advanced age and higher morbidity load increased the rate of dying and of being rehospitalized (model 1), decreased the rate of being discharged from hospital (models 1 and 2) and increased the rate of inactivation of IHC (model 2). WHF was an important risk factor associated with hospital readmission.

**Conclusion:**

Multi-state models enable a better identification of two patterns of HF patients. Once adjusted for age and comorbidity load, the WHF condition identifies patients who are more likely to be readmitted to hospital, but does not represent an increasing risk factor for activating ICU/IHC. This highlights different ways to manage specific patients’ patterns of care. These results provide useful healthcare support to patients’ management in real world context. Our study suggests that the epidemiology of the considered clinical characteristics is more nuanced than traditionally presented through a single event.

## Introduction

Heart failure (HF) prevalence steeply increases with age, from less than 1% in the population aged between 20 and 39 years, to more than 20% in individuals over 80 years [1, 2]. Population based studies report that one-year mortality rate ranges from 35% to 40% [3–7] and more than 50% of patients are re-admitted to hospital between six and twelve months after the first diagnosis [8, 9].

In this epidemiological scenario, the elderly with HF are representative of the growing segment living longer with chronic health conditions prone to multiple transitions from hospital to home that negatively affect their quality of life and consume substantial healthcare resources [10].

Given this public health issue, there is an urgent need to transform HF healthcare systems in order to improve evidence-based practice and create seamless care systems. To this end, a deeper understanding of clinical factors contributing to lengthen hospital stays and to increase multiple readmission rates to both hospitals and community services is essential.

However, most of the previous analyses attempting to identify those patients who are at risk of readmission, focused their attention only on the single re-hospitalization and failed to incorporate multiple hospital readmissions and admissions in community services that characterize a chronic HF disease [11–14]. In particular, knowledge gaps of these aspects remain on HF patients derived from the real world. In this respect, administrative data has been acquiring an important role over the years, since it provides useful information about the patient’s status and pattern of care, especially when the data is fully integrated with clinical data generated as part of routine patient care [15, 16].

Hence, an application of a model that considers all possible clinical pathways and assesses their dependence on important clinical covariates may be required. In this regard, a multi-state model provides a relevant modelling framework for event history data on chronic condition of HF patients [17–19].

A first attempt to apply these kinds of models to HF patients’ clinical evolution using administrative data, is described in Postmus et al. [20], in Bakal et al. [21] and in Ieva et al. [22]. However, all these studies focused only on hospitalizations of HF patients. In this work, we propose the application of two different multi-state models by considering administrative data integrated with electronic clinical data to investigate the impact of patients’ risk profiles on multiple hospitalization readmissions, home care (IHC) activations or intermediate care units (ICU) readmissions and death.

## Materials and methods

### Ethics statement

The current study involves the extraction of clinical records and administrative data produced as part of routine medical care. All participating sites are required to comply with the local regulatory and privacy policies and data is collected in an anonymous form. The informed consent is obtained under the institutional review board policies of hospitals administration.

### Study setting and cohort

Between January 2009 and December 2014, patients hospitalized and discharged with HF diagnosis in the Trieste area (the North-Eastern regional district in Italy) were recruited. HF diagnosis included ICD-9CM codes for HF (428:x) and hypertensive HF (402:01, 402:11, 402:91) according to the National Outcome Evaluation Program (in Italian PNE -Programma Nazionale Esiti) made by the national agency of Regional Health-Care Services (in Italian AGENAS—AGEnzia NAzionale per i servizi Sanitari regionali). Patients were classified as worsening heart failure (WHF) or de novo on the basis of the presence, or absence, of at least one HF hospitalization in the 5 years preceding the index admission, which is the first admission during the study period.

### Database

To select patients and clinical variables, administrative regional health data of the Friuli Venezia Giulia Region, integrated with data derived from the Outpatient and Inpatient Clinic E-chart (Cardionet^®^) in Trieste was used.

The E-Chart includes medical information collected by cardiologists during routine clinical practice, like diagnostic codes, laboratory tests, procedures, and drugs prescriptions sorted out using electronic indeces. The E-Chart also provides electronic access to folders, i.e. clinical consultations, emergency department visits, instrumental procedures, laboratory analyses and hospital admissions. Medical records are routinely reviewed by clinicians in each clinical evaluation to update medical history, diagnostic procedures and treatment. The E-Chart has been fully integrated in the Regional Data warehouse that contains regional databases, such as the Registry of Births and Deaths, the Hospital Discharge data, the District Healthcare Services (like IHC or ICU) and the Public Drug Distribution System.

This integrated database constitutes the Trieste Observatory of Cardiovascular Diseases. It covers the Trieste population, i.e., 237,000 inhabitants. Administrative data is collected in line with the rules of the National Health Service (NHS) in Italy that provides universal health coverage and collects several pieces of information including:

1. demographic data of all beneficiaries of NHS care (virtually the whole resident population);
2. diagnosis at discharge from public hospitals;
3. outpatient drug prescriptions, reimbursable by the NHS.

The administrative censoring date was September 30th, 2015. The original dataset was composed of 4,921 patients. We eliminated 17 patients for technical reasons. Thus, the final cohort comprised 4,904 patients. In order to protect privacy, information retrieved from the different databases was linked via a single anonymous identification code by institutional technical staff. The reverse process is not possible since the generation code table is not available to the authors.

The percentage of missing data in the administrative data warehouse was less than 0.1%. Of note is the fact that population-based research of cardiovascular diseases is feasible in the area of Trieste because public health care system is quite prevalent (87% of all cardiovascular ambulatory clinical evaluations, based on administrative reports).

This is one of the first attempts in Italy, to the best of our knowledge, at systematic integration between administrative and Electronic Health Recording Systems at regional level.

### Clinical variables and comorbidities

For each cohort member, data included gender and age, length of stay, department of admission and discharge, diagnostic code at discharge, stay in Emergency/Intensive Care Units during the hospitalization, cardiological evaluation before hospitalization (when performed), laboratory tests, echocardiographic Left Ventricular Ejection Function (LVEF) (when performed). The Charlson comorbidity index [23] was calculated using hospital diagnosis based on ICD-9CM that occurred within five years prior to the first admission and integrated with laboratory data and diagnosis recorded at the first admission. In particular, for the diagnosis of diabetes mellitus we integrated information about glycosylated haemoglobin on admission and the recorded diagnosis of diabetes mellitus in the previous 5 years. Similarly, to determine the presence of a chronic kidney disease, we integrated the creatinine value on admission to compute the estimated glomerular filtration rate (eGFR) < 60 ml/min (with the CKD-EPI formula) with the reported diagnosis of chronic kidney disease in the previous 5 years [24].

Other methods for identifying the comorbidity degree in elderly patients are available (see, among others, the Cumulative Illness Rating Scale—CIRS [25]), but they are not suitable for the kind of data in use, since not all the quantities requested in such indices are measured in our observational study.

### Outcomes

Study outcomes of interest included death for any causes, all-cause rehospitalization, and transitions in IHC/ICU. Death data was collected from the regional Registry of Birth and Deaths. All-cause hospitalizations and admissions in IHC/ICU were collected respectively from the Hospital Discharge Registry and the District Healthcare Services database. The principal discharge diagnosis for each hospitalization was assessed using primary ICD-9CM code. Each cohort member was followed from the starting date (i.e. discharge from the index admission) until the end of the study or the date of death, whichever came first.

### Statistical analysis

Patient characteristics are presented as numbers and percentages for categorical variables and means with standard deviations for continuous ones, or medians with interquartile ranges (IQRs) where relevant. WHF vs. de novo and people discharged from the Cardiological Ward (CW) vs. others are also compared. All these descriptions are reported in Tables 1, 2 and 3.

**Table 1:** Descriptive analysis of the whole cohort.

Variable	
N (%)	4,904
Male (%)	2,229 (45)
Mean age (sd)	80.74 (10.24)
Cancer (%)	504 (10)
Pulmonary disease (%)	1,316 (27)
Diabetes* (%)	1,468 (30)
Renal disease* (%)	2,973 (61)
LVEF^⋆^ (Q1;Q2)	53 (36; 64)
LVEF^⋆^ ≥ 50 (%)	1,786 (57)
LVEF^⋆^ < 50 (%)	1,363 (43)
Median Charlson index (Q1;Q3)	2 (1;4)
Pre hospitalization cardiological evaluation (%)	1,837 (37)
WHF (%)	775 (16)
Admission in CW (%)	1,136 (23)
Median LOS first event (Q1;Q3)	8 (5;14)
Death (%)	2,714 (55)
In hospital death (%)	1,444 (29)
Death in a year (%)	373 (8)
Median follow-up months (Q1;Q3)	26.2 (11.1; 48.0)
Median follow-up months survivors (Q1;Q3)	44.7 (26.7;62.7)
^⋆^LVEF missing values (%)	1,755 (36)

Cohort demographics for all patients. Each value refers to the first event, the index hospitalization. Comorbidities marked with * arise from integration of the administrative data and the laboratory tests results, as explained in Section Clinical variables and comorbidities.

**Table 2:** Descriptive analysis of rehospitalizations for any cause.

Variable	Patients with only index hospitalization	Patients with one rehospitalization	Patients with two rehospitalizations	Patients with three rehospitalizations	P-value
N (%)	1,423 (29)	1,131 (23)	740 (15)	554 (11)	
Male	634 (45)	498 (44)	332 (45)	254 (46)	0.920
Mean age (sd)	81.02 (11.36)	82.47 (9.99)	82.62 (10.24)	82.71 (9.12)	0.003
Cancer (%)	143 (10)	163 (14)	133 (18)	98 (18)	< 0.001
Pulmonary disease (%)	304 (21)	311 (28)	250 (34)	220 (40)	< 0.001
Diabetes* (%)	394 (28)	315 (28)	237 (32)	201 (36)	< 0.001
Renal disease* (%)	843 (59)	678 (60)	483 (65)	376 (68)	< 0.001
LVEF^⋆^ (Q1;Q2)	51 (34;63)	54 (35.25;64)	54 (39;63)	55 (39;64)	0.009
LVEF^⋆^ ≥ 50 (%)	447 (53)	434 (57)	314 (59)	256 (61)	0.047
LVEF^⋆^ < 50 (%)	391 (47)	328 (43)	222 (41)	163 (39)	0.047
Median Charlson index (Q1;Q3)	2 (1;3)	3 (1;4)	3 (2;5)	4 (2;5)	< 0.001
Pre hospitalization cardiological evaluation (%)	476 (33)	487 (43)	360 (49)	303 (55)	< 0.001
WHF (%)	107 (8)	136 (12)	118 (16)	101 (18)	< 0.001
Admission in CW (%)	348 (24)	157 (14)	79 (11)	55 (10)	< 0.001
Median LOS last hosp. (Q1;Q3)	8 (4;14)	8 (5;15)	9 (5;16)	9 (5;16)	< 0.001
Death (%)	603 (42)	634 (56)	447 (60)	336 (61)	< 0.001
In hospital death (%)	254 (18)	378 (33)	254 (34)	206 (37)	< 0.001
Death in a year (%)	255 (18)	193 (17)	161 (21)	111 (20)	0.051
HF hospitalization (%)	1,420 (100)	242 (21)	132 (18)	115 (21)	< 0.001
Median follow-up months (Q1;Q3)	18.4 (2.2;34.9)	10.7 (0.53;25.1)	4.60 (0.50;20.4)	3.30 (0.43;20.3)	< 0.001
Median follow-up months survivors (Q1;Q3)	29.5 (19.7;47.9)	26.5 (18.3;41.6)	22.7 (16.4;36.0)	22.4 (16.6;35.8)	< 0.001
^⋆^ LVEF missing values (%)	585 (41)	369 (33)	204 (28)	135 (24)	

Cohort demographics along the hospitalizations. Each value refers to the last hospitalization recorded. Comorbidities marked with * arise from integration of the administrative data (hospital discharge papers) and the laboratory tests results, as explained in Section Clinical variables and comorbidities. Wilcoxon tests are done for continuous covariates and tests on equal proportions are done for binary covariates.

**Table 3:** Descriptive analysis of ICU/IHC events.

Variable	Patients with at least one ICU or IHC events	Patients with no ICU or IHC events	P-value
N (%)	2,837 (58)	2,067 (42)	
Male	1,207 (43)	1,022 (49)	< 0.001
Mean age (sd)	81.15 (9.11)	80.19 (11.60)	0.383
Cancer (%)	299 (11)	205 (10)	0.509
Pulmonary disease (%)	799 (28)	517 (25)	0.015
Diabetes* (%)	895 (32)	573 (28)	0.004
Renal disease* (%)	1,757 (62)	1,216 (59)	0.030
LVEF^⋆^ (Q1;Q2)	55 (39;65)	50.50 (33;63)	< 0.001
LVEF^⋆^ ≥ 50 (%)	1,104 (60)	682 (52)	< 0.001
LVEF^⋆^ < 50 (%)	727 (40)	636 (48)	< 0.001
Median Charlson index (Q1;Q3)	2 (1;4)	2 (1;3)	< 0.001
Pre hospitalization cardiological evaluation (%)	1,080 (38)	757 (37)	0.316
WHF (%)	496 (17)	279 (14)	< 0.001
Admission in CW (%)	562 (20)	574 (28)	< 0.001
Median LOS first event (Q1;Q3)	14 (7;32.5)	7 (4;13)	< 0.001
Death (%)	1,610 (57)	1,104 (53)	0.022
In hospital death (%)	707 (25)	737 (36)	< 0.001
Death in a year (%)	150 (5)	223 (11)	< 0.001
Number of HF hospitalizations	1.72 (1.38)	1.33 (0.79)	< 0.001
Number of overall hospitalizations	3.00 (2.73)	1.92 (1.48)	< 0.001
Median follow-up months (Q1;Q3)	31.7(16.0;51.6)	19.4 (3.6;42.5)	< 0.001
Median follow-up months survivors (Q1;Q3)	47.1 (29.3;48.0)	40.4 (24.7;58.7)	< 0.001
^⋆^LVEF missing values (%)	1,006 (35)	749 (36)	

Cohort demographics for patients experiencing/not experiencing ICU and/or IHC event. Each value refers to the first event, the index hospitalization. Comorbidities marked with * arise from integration of the administrative data and the laboratory tests results, as explained in Section Clinical variables and comorbidities. Wilcoxon tests are done for continuous covariates and tests on equal proportions are done for binary covariates.

**Fig 1:**
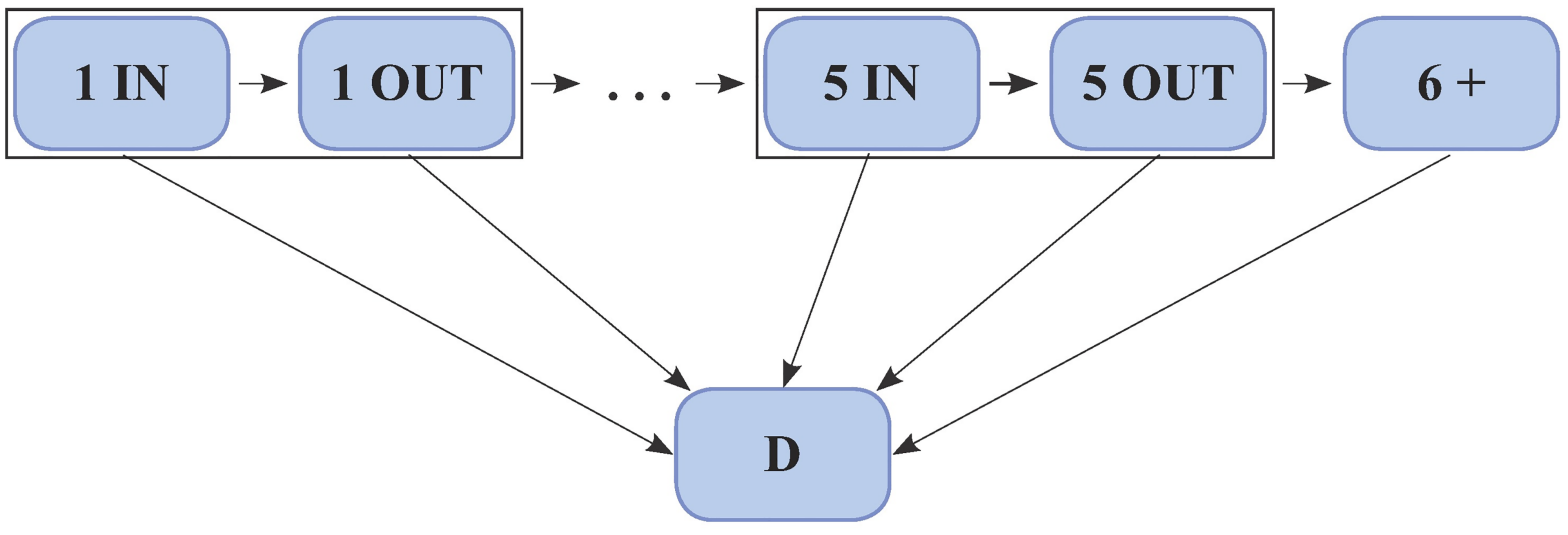
Diagram of model 1. The first five hospitalizations are considered. “In” stands for admission in hospital, “Out” for discharge from hospital and “D” for death. Patients with first admission for HF are considered. No distinction has been done between rehospitalization for heart failure or for any cause.

The first model (hereafter referred to as model 1) is shown in Fig 1. It replicates a dynamics similar to the one described in [22] for repeated hospitalizations only (we are omitting community services in this case), i.e. a multi-state model fitting a cox-type regression for each transition. It provides a convenient description of the admission-discharge dynamics, pointing out which covariates act in certain transitions and how they affect the relative risk as well as the risk (i.e. the instantaneous probability) of moving from one state to another. This model accounts for patient specific risk profile (distinguishing covariates acting on different transitions) as well as clinical information. In order to tackle the issue of a possible over-dispersion due to unobserved variables at patient level, we tested the first model including a gamma distributed frailty term. This approach led to a significant frailty, but the estimated variance of the frailty was 0.03. So, for the sake of model simplicity, we decided to continue the analysis without the frailty term.

**Fig 2:**
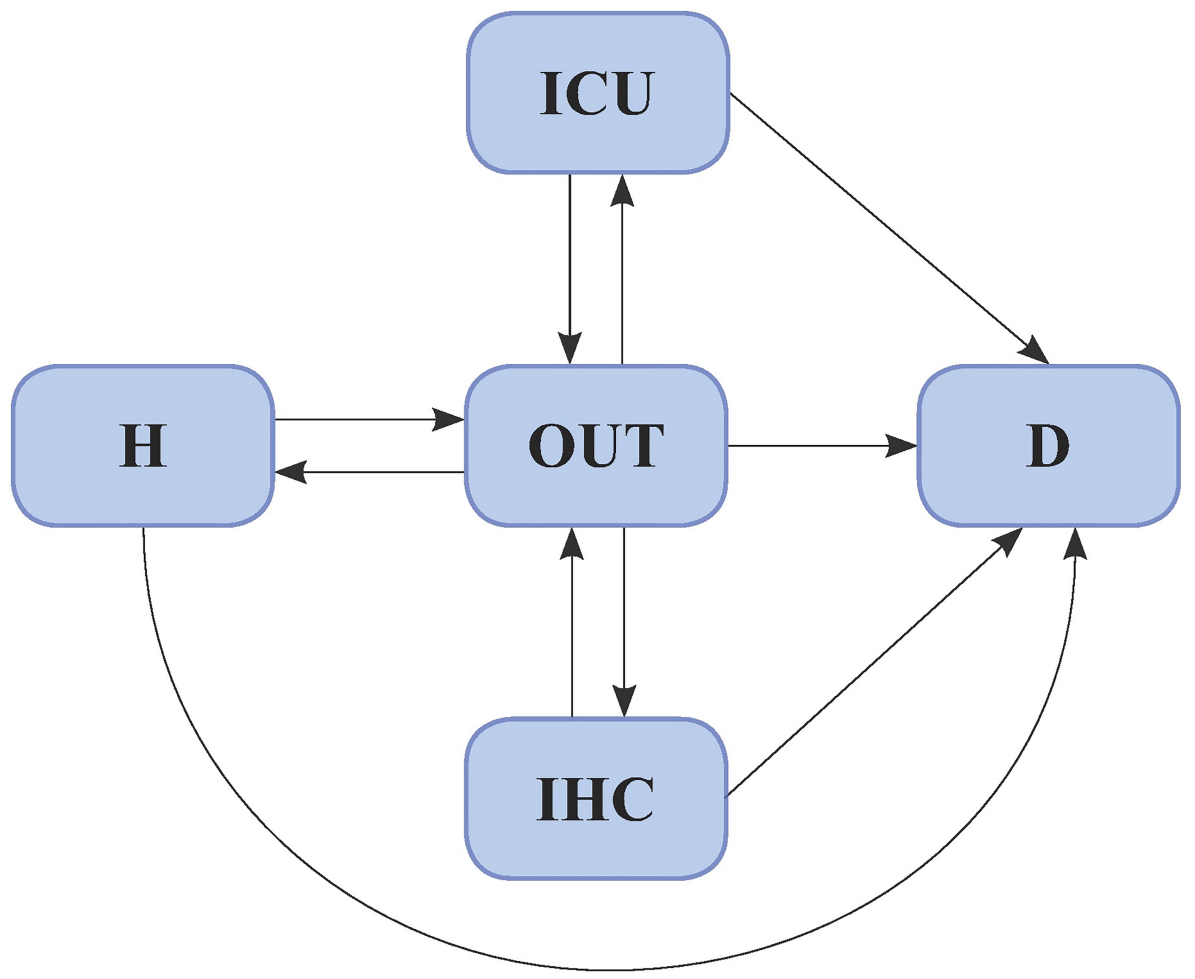
Diagram of model 2. The state space is made by all the possible events described in the dataset: admission to hospital (H), to ICU or IHC, discharge from any state (OUT) and death.

The second model (hereafter referred to as model 2) is shown in Fig 2. It is still a multi-state model where patients are assumed to be in one of the following five states: in hospital, in ICU, in IHC, OUT (of hospital, or ICU or IHC) and dead. Through this model, we seek to detect the impact of patient characteristics on the risk of moving within these states.

Both models include the adverse outcome of death, namely “D” in Figs 1 and 2, as absorbing state. The death of a patient (corresponding to all the possible transitions to “D” in Figs 1 and 2) is a competing event with respect to all the other transitions. For example, in models 1 and 2 a patient, who is in hospital for the first time, is eligible to be discharged or to die. The main difference between the two models concerns the competing transitions from the discharge event (OUT). Indeed, while in model 1 a patient can be readmitted in hospital or die, in model 2 a patient can be readmitted to hospital or in a community service as IHC or ICU or die.

The outcomes of these models are reported in Tables 4 and 5 as transition-specific hazard ratios (together with the corresponding confidence intervals). We tested both models with three sets of covariates, differentiating them from the way the multi-morbidity load is expressed: in particular, in the first covariate set, the Charlson index was considered. In the second set we included the simple sum of the number of comorbidities, and in the third set we included separately some of the most dangerous comorbidities (tumor, diabetes, renal disease and pulmonary disease). In all the three sets we considered age (in all transitions), gender (in readmission transitions), WHF condition (in all readmission and transitions to death) and finally Cardiological Ward stay (in the hospital discharge and transitions to death). The choice of these covariate patterns was informed by previous attempts, testing the full model with all covariates in all transitions. All the three test settings gave similar outputs. So, for the sake of simplicity, we decided to focus on the models containing the Charlson index.

**Table 4:** Hazard rates (and corresponding 95% CIs) for transitions of model 1.

	Age (5 years)	CH index (2 points)	WHF	Male vs Female	CW
Readmission to H_2_	1.07 (1.05, 1.09)	1.19 (1.15, 1.23)	1.34 (1.22, 1.46)	1.05 (0.98, 1.13)	
Readmission to H_3_	1.05 (1.02, 1.07)	1.16 (1.12, 1.20)	1.38 (1.25, 1.53)	1.02 (0.93, 1.11)	
Readmission to H_4_	1.04 (1.02, 1.07)	1.16 (1.11, 1.21)	1.30 (1.16, 1.46)	1.04 (0.94, 1.16)	
Readmission to H_5_	1.04 (1.00, 1.08)	1.18 (1.12, 1.25)	1.39 (1.21, 1.59)	0.92 (0.81, 1.05)	
Readmission to H_6+_	1.00 (0.96, 1.05)	1.12 (1.05, 1.19)	1.22 (1.04, 1.43)	1.07 (0.92, 1.24)	
Discharge from H_1_	0.99 (0.97, 1.01)	0.91 (0.89, 0.94)			1.59 (1.47, 1.72)
Discharge from H_2_	0.95 (0.93, 0.97)	0.90 (0.88, 0.93)			1.39 (1.25, 1.53)
Discharge from H_3_	0.98 (0.95, 1.00)	0.99 (0.95, 1.02)			1.49 (1.30, 1.70)
Discharge from H_4_	0.96 (0.93, 0.99)	0.93 (0.89, 0.98)			1.39 (1.17, 1.64)
Discharge from H_5_	1.01 (0.97, 1.05)	0.94 (0.89, 0.99)			1.60 (1.30, 1.97)
Death in H_1_	1.59 (1.45, 1.75)	1.25 (1.13, 1.39)	0.82 (0.59, 1.14)		0.33 (0.14, 0.76)
Death in H_2_	1.30 (1.21, 1.40)	1.14 (1.05, 1.24)	0.87 (0.67, 1.14)		0.60 (0.37, 0.97)
Death in H_3_	1.34 (1.23, 1.46)	1.21 (1.10, 1.33)	0.86 (0.64, 1.16)		0.67 (0.36, 1.25)
Death in H_4_	1.35 (1.22, 1.49)	1.08 (0.97, 1.21)	0.90 (0.65, 1.23)		0.56 (0.27, 1.16)
Death in H_5_	1.32 (1.17, 1.49)	1.10 (0.96, 1.27)	1.00 (0.69, 1.45)		0.44 (0.14, 1.41)
Death in H_6+_	1.27 (1.19, 1.34)	1.19 (1.11, 1.27)	0.89 (0.74, 1.08)		0.70 (0.46, 1.06)
Death out of H_1_	1.50 (1.39, 1.62)	1.39 (1.27, 1.53)	0.60 (0.43, 0.86)		0.60 (0.40, 0.90)
Death out of H_2_	1.55 (1.42, 1.70)	1.39 (1.26, 1.54)	0.88 (0.63, 1.23)		0.78 (0.49, 1.23)
Death out of H_3_	1.33 (1.21, 1.46)	1.36 (1.22, 1.52)	0.69 (0.47, 1.01)		0.44 (0.22, 0.88)
Death out of H_4_	1.51 (1.34, 1.71)	1.23 (1.06, 1.43)	0.72 (0.46, 1.13)		0.72 (0.34, 1.51)
Death out of H_5_	1.16 (1.02, 1.33)	1.22 (1.02, 1.46)	0.91 (0.56, 1.50)		1.11 (0.54, 2.29)

**Table 5:** Hazard rates (and corresponding 95% CIs) for transitions of model 2.

	Age (5 years)	CH index (2 points)	WHF	Male vs Female	CW
Readmission to H	1.04 (1.03, 1.05)	1.19 (1.17, 1.21)	1.30 (1.24, 1.36)	1.04 (1.00, 1.08)	
Readmission to ICU	1.19 (1.16, 1.22)	1.16 (1.13, 1.20)	0.92 (0.83, 1.02)	0.76 (0.69, 0.83)	
Readmission to IHC	1.05 (1.03, 1.06)	1.14 (1.12, 1.17)	1.03 (0.97, 1.10)	0.99 (0.94, 1.05)	
Discharge from H	0.98 (0.97, 0.99)	0.94 (0.93, 0.95)			1.46 (1.39, 1.53)
Discharge from ICU	0.98 (0.95, 1.01)	1.03 (1.00, 1.07)			
Inactivation of IHC	1.04 (1.03, 1.06)	1.18 (1.16, 1.21)			
Death in H	1.35 (1.30, 1.40)	1.20 (1.16, 1.25)	0.89 (0.79, 1.01)		0.52 (0.39, 0.68)
Death in ICU	1.09 (0.99, 1.20)	1.53 (1.40, 1.67)	0.72 (0.51, 1.01)		
Death in IHC	1.42 (1.27, 1.59)	1.15 (1.00, 1.31)	0.94 (0.62, 1.42)		
Death OUT	1.44 (1.38, 1.51)	1.25 (1.18, 1.31)	0.81 (0.68, 0.96)		0.83 (0.65, 1.05)

All the analyses were carried out using the free software R [26]. Specifically, packages survival [27, 28], mstate [19, 29, 30] and msm [18] were used for processing data and implementing the models. Codes are available from the authors upon request.

## Results

A total of 4,904 patients hospitalized with primary HF diagnosis between January 1st, 2009 and December 31st, 2014 were identified. The characteristics of the whole cohort are described in Table 1. The mode of clinical presentation of patients was de novo HF involving 4,129 patients (84%), and WHF involving 775 patients (16%). Overall, the mean age was 81 years with a substantial proportion of female patients with significant background prevalence of non-cardiac comorbidities, see Table 1. 2,923 (71%) out of the 4,129 de novo patients had a previous hospitalization for any cause. Indeed, more than half of the cohort (61%) had a renal disease and the median of LVEF, when recorded, was 53% (30% with LVEF < 40%; 13% with LVEF 40–49%; 57% with LVEF ≥ 50%). Comorbidity burden was high, with the median of Charlson index of 2 (40% with Charlson index ≥ 3). The rate of admission in cardiological ward (CW) was 23% at the first hospitalization. The median follow-up was 26 months. During five years of observation, 2,714 deaths out of 4,904 patients (55%) were recorded, 2,115 were de novo patients, out of 4,129 (51%), whereas 599 were WHF, out of 775 (77%). Overall, 1,444 patients (29% of the cohort) died during a hospitalization. The majority of in-hospital deaths (1,037; 72%) occurred during hospitalization for any cause, whereas 407 (28%) during HF hospitalization. Overall, there was a high morbidity burden in the total amount of 23,665 events. 7,634 events (32%) of the total events were HF hospitalizations, 8,329 (35%) were hospitalizations for any cause, 2,303 (10%) were admissions in ICU and 5,399 (23%) were admissions in IHC. 1,316 (27%) de novo patients had only one hospitalization, and 520 (10% of the overall population) died after or during the first and unique event.

The clinical characteristics of the cohort, according to the number of hospitalizations for any cause (after the first index HF admission), are shown in Table 2. Overall, the progressive number of readmission rates was associated with aging, thus, increasing the WHF percentage and comorbidity burden. Notably, in-hospital mortality rate increased by nearly half with re-hospitalization rates rising from 18% to 37%.

The hazard rates, estimated in model 1, are recorded in Table 4. A significant effect of aging and increase of comorbidity burden on the re-hospitalization risk was observed (Table 4, first block from the top). Likewise, a relevant impact of the clinical condition of WHF was observed in all readmission rates. No significant role of gender emerged. In Table 4 (second block from the top), the impact of covariates on instantaneous probability of being discharged from hospital, i.e. shortening the Length-Of-Stay (LOS), is displayed. As expected, this probability was inversely related to age and Charlson index. Conversely, a direct relation with admission in cardiological ward was observed. When we considered the effect of covariates on risk of mortality related to readmission, the hospitalization in cardiological ward was protective up to the second hospitalization, while, after that, this effect was nullified (Table 4, third block from the top). Furthermore, aging and increase in the Charlson index were still associated with in and out of hospital deaths through all readmissions, whereas almost every adverse effect on death was observed for clinical condition of WHF (Table 4, fourth block from the top).

The second part of the analysis focused on including the outpatient care services. Descriptive characteristics of patients, with or without admission in ICU/IHC, are shown in Table 3. Compared with the patients without admission in ICU/IHC, patients referred for ICU/IHC were predominantly female (57% vs. 51%), with higher prevalence of non-cardiac comorbidities and a clinical history characterized by frequent hospitalizations for any cause (3 vs. 2 the median values). Despite the fact that patients in ICU/IHC showed globally higher mortality rates, a lower in-hospital death occurred in this HF patients’ subset.

The impact of patients’ characteristics on the risk of moving among these transitions (model 2) is shown in Table 5. We observed that aging and higher Charlson index increased the risk of being readmitted to hospital, ICU and IHC (Table 5, first block from the top). When we considered the covariates effect on time spent in different states of model 2, aging process was directly related to time spent in hospital, while it was inversely related to time spent in IHC (Table 5, second block from the top). Furthermore, we noticed a higher risk of admission in ICU for female patients. WHF condition increased the risk of being readmitted to hospital (Table 5, first block from the top) and it behaved as a protective factor for death outside (Table 5, last block from the top). Model 1 indicates that the hospitalization in a cardiological ward was related to a lower in-hospital death rate and a shorter length of stay.

**Fig 3:**
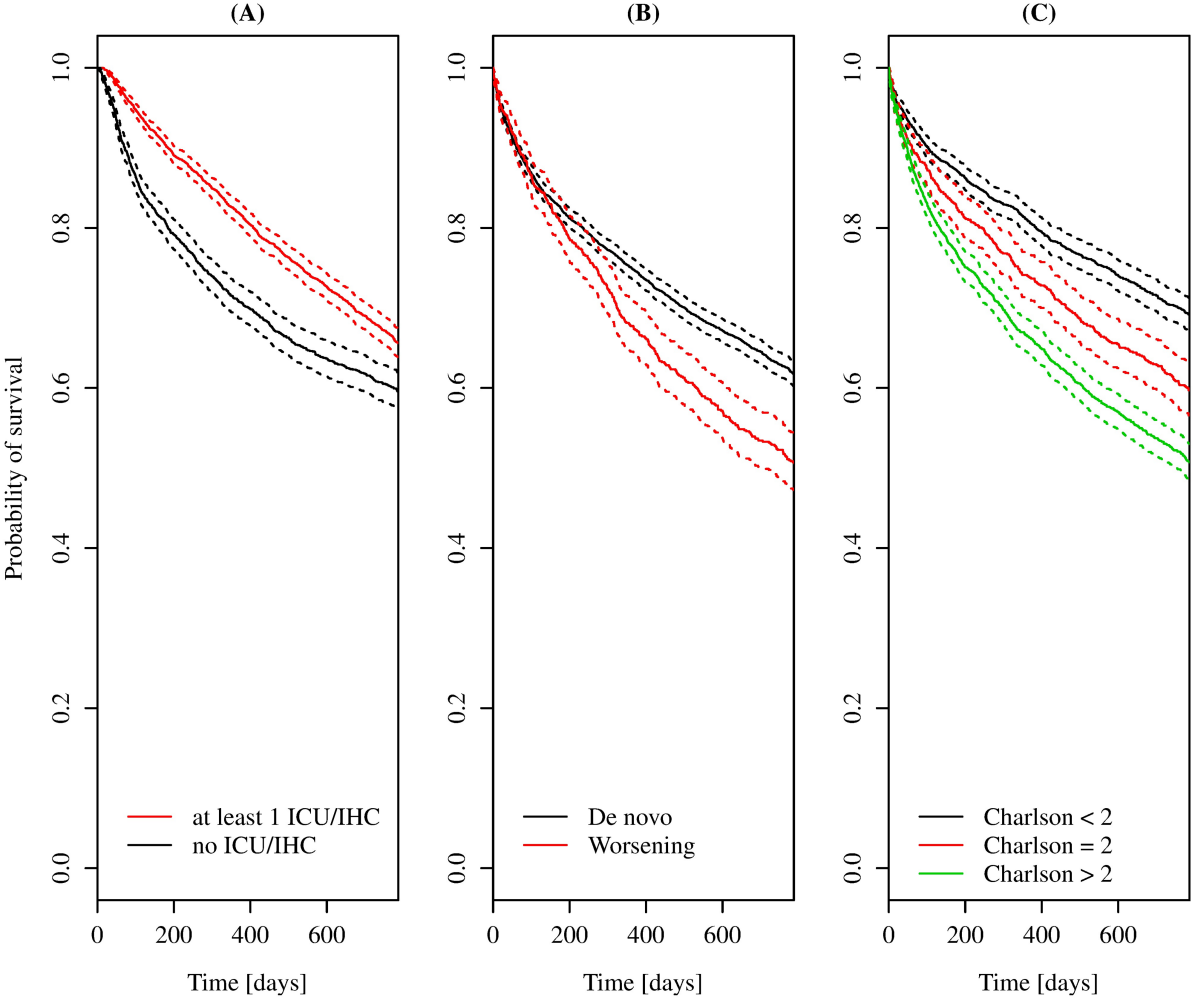
Kaplan-Meier curve for survival time of patients up to the median follow-up time. In panel (A), curves are stratified by the presence of at least one ICU or IHC in patient’s clinical history. In panel (B), curves are stratified by being de novo or WHF patients. In panel (C), curves are stratified by quartiles of the Charlson index.

In order to confirm the impact of the covariates on death, we investigated the Kaplan Meier curves of survival time. We distinguished between patients who were or were not admitted to community services (Fig 3A), between WHF vs. de novo patients (Fig 3B) and between patients with different Charlson index at their first event (Fig 3C). The last stratification was carried out according to Charlson index quartiles, recorded in Table 3. As expected, a higher Charlson index and WHF state were confirmed as important factors associated with high mortality risk. Finally, we pointed out that HF patients with at least one admission in IHC/ICU showed a significantly lower mortality risk up to the median follow-up time (Fig 3A).

## Discussion

Our study complements and expands the findings of previous observational studies that assessed the adverse outcome based only on single event in HF patients. For the first time, we demonstrate the effect of certain clinical conditions in a community setting on multiple readmissions by including intermediate care services (IHC/ICU). These findings significantly enhance our understanding of the clinical pattern of patients with HF for adverse prognosis and have implications for their management approach. From a policy perspective, identification of patients at high risk of multiple readmissions must include the implementation of appropriate preventable intervention strategies [31].

Although in HF populations the single event is appropriate for characterizing prognosis, nowadays the greatest interest lies in the identification of global risk profile of HF patients with clinical complexities that lead to multiple readmissions.

Indeed, our approach focuses on multiple events. Patients with high risk profile are identified according to their transition patterns in the multi-state models in order to identify clinical factors associated with different transitions (i.e. admission, discharge and death). To date, multi-state models have been one of the most applied statistical tools for estimating both competing and recurrent events, such as hospitalizations among patients suffering from cancer, thus, allowing policymakers to anticipate the potential impact of preventive strategies [32].

For the first time Postmus et al. [20] applied a multi-state approach to HF events by including HF patients from a multicenter, randomized controlled trial, potentially limiting the applicability of the findings to cohorts of HF patients seen in clinical practice. Moreover, the authors included only the primary end-point of HF hospitalization, introducing the possibility of underestimating the overall comorbidity burden.

On the contrary, our interest focused on overall hospitalizations in real world setting rather than HF hospitalizations, in order to improve our knowledge on total morbidity burden of HF. This is crucial for a deeper understanding of morbidity burden of HF patients at community level and allowing healthcare providers to better allocate resources to HF services. Specifically, for the first time, we assessed the factors associated with intermediate states representing the admissions in IHC/ICU. Consistently with previous epidemiological studies focused on HF population [33–35], our cohort presented a high mean age (81 years) with a high comorbidity burden (Table 1). Patients with multiple readmissions were elderly, with higher comorbidity burden (Table 2). Mortality rates dramatically increased with multiple readmission achieving 61% in patients with three hospitalizations and 37% of in-hospital mortality. In our approach, it was clear how the presence of coexisting non cardiovascular conditions affected rehospitalizations of HF patients.

When assessing hazard rates with multi-state models by considering readmissions in hospital for any cause (model 1), we found that an increased age and comorbidity condition (identified by Charlson index) and WHF were all associated with higher rates of readmission (Table 4, first block from the top). However, only aging and increasing comorbidity load were confirmed as factors for higher, in and out, mortality rates (Table 4, last block from the top). The higher risk attributable to aging and comorbidity condition was in line with previous observations that underlined the association between comorbidity burden and mortality in chronic HF [36, 37]. Notably, the advanced age and Charlson index were inversely related to the probability of being discharged from hospital (Table 4, second block from the top).

Our results cannot be compared with previous analysis outcomes in HF patients, since the most part of other studies focus on single events and not on the whole joint dynamics of hospitalizations/clinical events and death, as it is in a multi-state model approach. This is the greatest power of such kinds of models: they enable us to successfully detect different impacts of different covariates on different transitions, all at the same time. Indeed, our study suggests that the epidemiology of the considered clinical characteristics is more nuanced than traditionally presented through a single event. Our results indicate the important role of patient’s risk profile on multiple readmissions.

Notably, the models presented in this study also take the patient’s disease progression one step further by introducing admissions in ICU/IHC. To the best of our knowledge, there are no previous studies that analyzed ICU/IHC admission in a community setting. In model 2, the aging and comorbidity conditions were strongly associated with both readmissions in hospital and ICU/IHC (Table 5, first block from the top). Moreover, female patients showed a higher risk of admission in ICU, probably linked to the widowed state (due to old age). Conversely, the WHF condition did not increase the risk of admission in ICU/IHC significantly, although it was still an important risk factor associated with hospital readmission. This latter result supported the fact that healthcare strategies implemented in high risk patients such as the elderly, with multiple and debilitating conditions, should continue to be provided for irrespective of their HF status. Despite their higher risk profile, patients admitted into ICU/IHC showed a lower risk of mortality up to the median follow up time (Fig 3A), thus, highlighting the potential effectiveness of ICU/IHC in HF management.

## Limitation and further development

At the end of this innovative application of multi-state models to the complex healthcare pattern of HF patients, we are aware of some limitations and also possible future development.

Firstly, since the LVEF echocardiographic measure was missing for a relevant quota of patients (36% at the index admission, Table 1) we chose not to include it in the analysis. Secondly, we did not use the prescribed pharmacological treatments at discharge stage as covariates in the models since the key information about adherence to therapy during follow up was lacking. Moreover, we are aware of the fact that Charlson index is not the best index to measure the comorbidity load in such an old cohort [25]. In this case, considering CIRS index (Cumulative Illness Rating Scale) would have been more appropriate, but we were not able to compute this index because some of the required variables (i.e. psychiatric conditions) are not routinely measured in the administrative records of Hospital Discharge data, neither in the cardiological Outpatient and Inpatient Clinic E-chart.

To conclude, we foresee the inclusion of LVEF, pharmacological treatment and CIRS index in these models as possible further developments. Moreover, the investigation of a possible relation between the admission in ICU/IHC and the number of hospitalizations can be a target to be addressed in future works.
